# Impact of manual therapy on body posture-3-D analysis with rasterstereography – pilotstudy

**DOI:** 10.1186/s13005-024-00450-0

**Published:** 2024-09-13

**Authors:** Alessia Celine Harhoff, Tobias Pohl, Christine Loibl, Werner Adler, Martin Süßenbach-Mädl, Johannes Ries, Anna Seidel, Manfred Wichmann, Ragai-Edward Matta

**Affiliations:** 1 Department of Prosthodontics, Erlangen University Hospital, Glueckstrasse 11, Erlangen, 91054 Germany Province Bavaria; 2 Department of Medical Informatics, Biometry and Epidemiology, Friedrich-Alexander-University of Erlangen, Waldstrasse 6, Erlangen, 91054 Germany; 3 Physiotherapy Praxis Ganzheitliche Physiotherapie, Bismarkstrasse 26, Erlangen, 91054 Germany

**Keywords:** Body posture, Three-dimensional imaging, Manual therapy, Physiotherapy, Rasterstereography, Temporomandibular disorders, Temporomandibular joint

## Abstract

**Introduction:**

The relationship between posture and temporomandibular disease (TMD) is unclear. The aim of our study was to determine the influence of manual therapy (MT) on posture in TMD patients compared with healthy subjects.

**Material/method:**

After consideration of inclusion and exclusion criteria, 30 subjects were included. These were divided into two groups: group A comprised 15 healthy subjects and group B 15 patients with present proven TMD disease. Rasterstereographic images were taken at different times. Group A subjects were scanned twice within half a year and group B before initiation as well as after the first MT and after completion of the prescribed MT. The different posture variables were calculated using DIERS Formetric software.

**Results:**

To illustrate the differences between the two groups, 10 different postural variables were examined. Significant differences between the two groups were observed in pelvic tilt, surface rotation, and kyphotic apex. Pelvic tilt: mean = 7.581, *p*-value = 0.029; surface rotation: mean = 3.098, *p* = 0.049; and mean kyphotic apex = 11.538 and 11.946, respectively, with *p*-values of 0.037 and 0.029, respectively.

**Conclusion:**

MT leads to a change in posture in TMD patients. This could influence the course of TMD treatment.

## Introduction

The question of a relationship between occlusion and posture and the significance of temporomandibular dysfunction (TMD) in this constellation is a much discussed issue in dentistry. While some studies support the hypothesis of an interplay between these variables, many of these studies lack the necessary evidence [1–5]. A potential interrelationship is very interesting for dentistry, as TMJ disorders are the second most common type of musculoskeletal disorders [6]. Temporomandibular dysfunction (TMD) is defined by the American Academy of Orofacial Pain (AAOP) as an umbrella term for musculoskeletal and neuromuscular diseases affecting the temporomandibular joint (TMJ), masticatory muscles, and other involved tissues, whose signs and symptoms can be multifaceted and include impairments in speech, mastication, and other orofacial functions [7–9].

TMD is a condition that, like many other pain syndromes, is not definitively understood. This is due to the frequent lack of correlation between TMD-related pain and definitive pathophysiological evidence, such as degenerative lesions or tissue damage [10]. Based on current knowledge, TMD appears to be a multicausal heterogeneous event of varying physical and psychological aetiologies [11]. Intra-articular factors such as inflammatory diseases, dislocations of the articular disc, and degenerative processes are assumed to be risk factors. Furthermore, extra-articular structures such as masticatory muscles and ligaments or cervical spine disorders in conjunction with altered cervical muscle tone can be considered as causes. Various authors have also assumed a causal relationship between postural problems and TMD [12–16]. In addition, psychosocial stressors have been discussed as a cause of TMD-related pain, especially pain of the masticatory muscles or altered pain processing of the central nervous system [17–19].

Epidemiological studies show that about 5% to 15% of the population is affected by TMD, and about half to one-third of TMD patients seek treatment [6, 20]. In this context, the frequency and severity of TMD increases during the period from the age of 20 to 40. In recent decades, studies have found a tendency for TMD disorders to shift to earlier ages [21, 22]. Women are affected significantly more often (factor of 2:1 to 4:1) [6, 23, 24] than men [21, 25]. Symptoms of temporomandibular disorders often include acute muscular pain during jaw movements, a tenderness or pain sensation around the temporomandibular joint, and limitations or deviations in the range of motion (ROM) of the mouth opening. Other symptoms may include joint noise during movement or a sensation of TMJ blockage [20, 26, 27]. In addition, TMD patients often report pain during mastication, as well as accompanying headache, shoulder pain, and neck pain [28].

Due to the multifactorial background of TMD, therapy can be multidisciplinary. Treatments for TMD are non-invasive or invasive. In a step-by-step scheme, TMD therapy can be started individually, depending on the cause, and intensified over its course. Regardless of the cause of the TMD, a non-invasive therapy is initially chosen. If there is no improvement, the therapy can then be supplemented or intensified by drug treatment. If the primary conservative treatment options are not successful, a surgical therapy approach in the form of arthrocentesis or TMJ surgery can be considered. The surgical procedure should increase from minimally invasive to invasive [29, 30].

Currently, a variety of non-invasive treatment approaches are available. The most common ones are described by various studies. These include the dental therapeutic approach using oral splint therapy [31, 32], as well as different physiotherapeutic techniques that include manual therapy, therapeutic mobilizations, self-exercises [33–37], and massage therapies [38], or transcutaneous electrical nerve stimulation (TENS) treatments [36, 39, 40]. Other studies focus on behavioural therapies and multimodal treatments, which combine stress reduction and relaxation exercises, among others [20, 41]. In addition, drug treatment options are also among the non-invasive approaches and may consist of, for example, administration of analgesics, muscle relaxants, or antidepressants [10, 30]. Non-invasive treatment methods are followed by minimally invasive ones in a stepwise scheme. These include direct injection of botulinum toxin into the associated TMJ muscles [42, 43] or performing arthrocentesis [44, 45]. In the most severe cases of TMJ damage, triggered for example by inflammation or trauma, invasive treatment options are used. The destroyed TMJ is surgically replaced by an implant [46, 47].

A frequently used primary conservative physiotherapeutic treatment technique is manual therapy (MT) [35, 48]. MT is used by physiotherapists to restore the range of motion (ROM, the amplitude of movement), to reduce local pain, generally relieve pain, and stimulate proprioception. MT is also used to dissolve tissue adhesions. Significant pain reduction can be achieved by mobilizing the jaw and upper cervical joints and cervical spine and by stretching the masticatory and neck muscles, as well as by passive movements performed by the therapist. In addition, MT increases ROM through mobilization techniques and muscle relaxations [33, 36, 49–51].

In previous studies, the influence of various non-invasive physiotherapeutic approaches, such as manual therapy, massage techniques, or relaxation and mobilization exercises, was demonstrated to significantly increase the maximum mouth opening (i.e., maximize ROM) and reduce pain in TMD patients [35, 38, 50–52]. According to Gomes et al., this effect occurred after only 4 weeks of treatment [38]. In a study by Furto et al., a reduction in pain was observed after only 2 weeks, through manual therapeutic treatment in combination with mobilization exercises of the TMJ [35].

A reliable, non-invasive method for three-dimensional (3D) analysis of spinal morphology is rasterstereography. This was developed in the 1980s by Drerup and Hierholzer and allows 3D reconstruction of the thoracic and lumbar spine. This technology is a fast, non-contact, and radiation-free method that calculates a 3D surface using image sequences [53]. Linear rasterstereography, based on photometry, provides a 3D representation of the patient's dorsal profile. Here, a beam of light is emitted from a projector, often a laser, onto the dorsal surface of the patient and a line grid is projected onto the patient's back. Using computer software, the line curvatures can be registered by a camera and the surface relief can be determined by optical distance measurement using triangulation. The patient assumes their habitual posture at a standardized distance of 2 m from the projector. The measurement data is determined with the help of a video-optical device. The result is a 3D-visualized model of the spine [54, 55]. To achieve even more accurate measurements, 4D methodology was introduced by the manufacturer. The Formetric 4D analysis system is a commonly used video rasterstereographic system. Compared with the 3D method, this system additionally evaluates dynamic events (posture fluctuations, respiratory movements), taking into account all image sequences. With the 4D technology, the posture variance is reduced via a subsequent mean value calculation. According to the manufacturer's instructions for use, 12 images are recorded within 6 s and calculated together so that slight breathing and swaying movements do not influence the measurement data. The main advantages of this method are its non-invasive and radiation-free nature [56, 57].

The effects of the aforementioned multiple etiological components of TMD are uncertain and not yet fully understood. One of the factors influencing TMD could be the spine or posture. However, a correlation of posture and TMD has yet not been proven [4, 5, 58].

Studies that assume a correlation between TMD and body posture mainly demonstrate postural changes in body segments close to the head and neck because of the close anatomical relationship and neurophysiological mechanisms between the two regions TMJ and neck/head [16, 59, 60]. The authors' assumption here is based on the theory that the stomatognathic system and the craniocervical complex are connected by an interrelated neuromuscular system and form a functional unit [61]. The temporomandibular joint in particular creates muscular and ligamentous connections to the cervical spine and thus forms the link to the ‘craniocervical system’. The most important disorders of this functional unit, which often affect posture, are TMD [61].

Therefore, a change in posture appears to affect the position of the mandible and the activity of the surrounding musculature, thereby internally causing TMJ dysfunction. Alterations in either the cervical spine or the TMJ may therefore cause dysfunction of the other system [62, 63]. The muscle-fascia chains represent a fundamental element between the stomatognathic system and posture. Fascia permeates and surrounds the human body in the form of dense, fibrous connective tissue [64]. The fascial system is important not only because it can passively distribute tension in the muscles of the body when mechanically stimulated, but also because it contains mechanoreceptors and has an autonomic contractile ability to influence the tension of the fascia [61]. These tensions are transmitted along the muscle-fascia chain and thus influence the posture of the entire body [64, 65]. A functional relationship between the cervical spine and TMD has also been demonstrated by Piekartz and Lüdtke through the positive effect of MT treatment in one area on the other [66]. Several other studies have documented modifications in head extension, pelvic torsion, and changes in cervical lordosis in TMD patients [12, 15, 16, 67–70]. Because the head and neck muscles are closely related to the stomatognathic system, studies have been conducted to confirm that changes in head and body posture can have an adverse biomechanical effect on the TMJ and thus cause TMD. However, the lack of appropriate measurement methods and the lack of scientific evidence between occlusal and postural characteristics have so far disallowed any conclusion on the basis of the obtained data that an effect relationship exists [71, 72]. Mousatafa et al. were able to show that patients with fibromyalgia who received MT in the cervical spine areas, among others, had significant changes in postural parameters after 12 weeks as well as at 1 year in comparison with a control group [73].

Currently there is a lack of studies demonstrating the effect of MT on the rasterstereographic postural profiles of TMD patients. Accordingly, our pilot study was conducted with the aim of testing the null hypothesis, which states that manual therapy in patients diagnosed with TMD has no effect on their posture compared with that of a control group.

## Materials and methods

### Subjects

The patient collective for the present study was prospectively acquired in the period from September 2019 to March 2021, from the patient collective of Zahnklinik 2 of the University Hospital Erlangen-Nuremberg. Prior to the start of the study, permission for the study (number 233_19B) was granted by the Ethics Committee of the University Hospital Erlangen-Nuremberg, and the study was registered with the DKRS/ BfArM.

All methods were performed in accordance with the ethical standards of the aforementioned ethics committee and the Declaration of Helsinki of 1975 as revised in 2008.

Included in the study were 15 patients (7 women, 8 men) with TMD diagnosed at baseline and 15 subjects (8 women, 7 men) who were unlikely to have TMD according to the Jakstat and Ahlers TMD screening [74].

The mean age of the patients at the time of study participation was 37.7 years (range: 24–79 years). Within the control group, the mean age was 26.5 years (range: 23–35 years).

The diagnosis of TMD was based on the "TMD short findings" according to Jakstat and Ahlers (Table 1) [75]. This screening test is a TMD brief finding that evaluates six different variables, each with a yes/no response, based on which TMD is judged likely or unlikely. The six variables are: mouth opening asymmetric, mouth opening restricted, joint sounds, occlusal sounds, muscle palpation painful, and eccentricity traumatic. No other aids are necessary for the findings and the test is reliable, easy, rapid, and reproducible. As soon as at least two of the six questions are answered in the affirmative by the practitioner, a TMD is to be regarded as probable and a detailed functional analysis should follow [74].

**Table 1:** TMD short findings according to Jakstat & Ahlers

TMD short findings	Tick where applicable
Mouth opening asymmetrical	
Mouth opening restricted	
Joint noises	
Occlusal sounds	
Muscle palpation painful	
Eccentricity painful	
**TMD ○**	Unlikely (≤ 1)
	Likely (≥ 2)

Only patients who had not previously received a known TMD diagnosis and had not received appropriate treatment at any previous time were included in the study. Exclusion criteria included past TMD therapy and cases in which a definitive TMD diagnosis could not be established. Minor patients and patients with surgery of the TMJ and surrounding structures were also not included in the study.

Interested patients received an information sheet on study participation and implementation, as well as a data protection and informed consent form. These had to be signed prior to study participation. Informed consent to participate in the study was obtained from all patients.

### Manual therapy treatment

Participating patients received a prescription for six treatments of manual therapy and six treatments of heat packs, which were performed as double treatments (3 × 40 min). The manual therapy treatment sessions, each with a maximum duration of 40 min, took place over a period of 3 to 4 weeks, with an average interval of 7 days. The patients always received their therapy sessions from the same therapist, who is specially trained in the field of cranio-mandibular therapy and has long-standing professional experience. The treatments were performed according to a standardized procedure. Prior to treatment, all patients received an examination of the spine and orofacial structures by the treating therapist in order to plan the subsequent course of treatment. Treatment procedures included techniques for mobilizing the cervical joints and the TMJ, manipulation of these joints, tender–trigger point treatments, stretching of the musculature, and coordination and home exercises. The therapist selected the techniques and the type of treatment or exercise that she deemed, in her judgment, to be beneficial to the patient.

### Rasterstereography

**Fig. 1:**
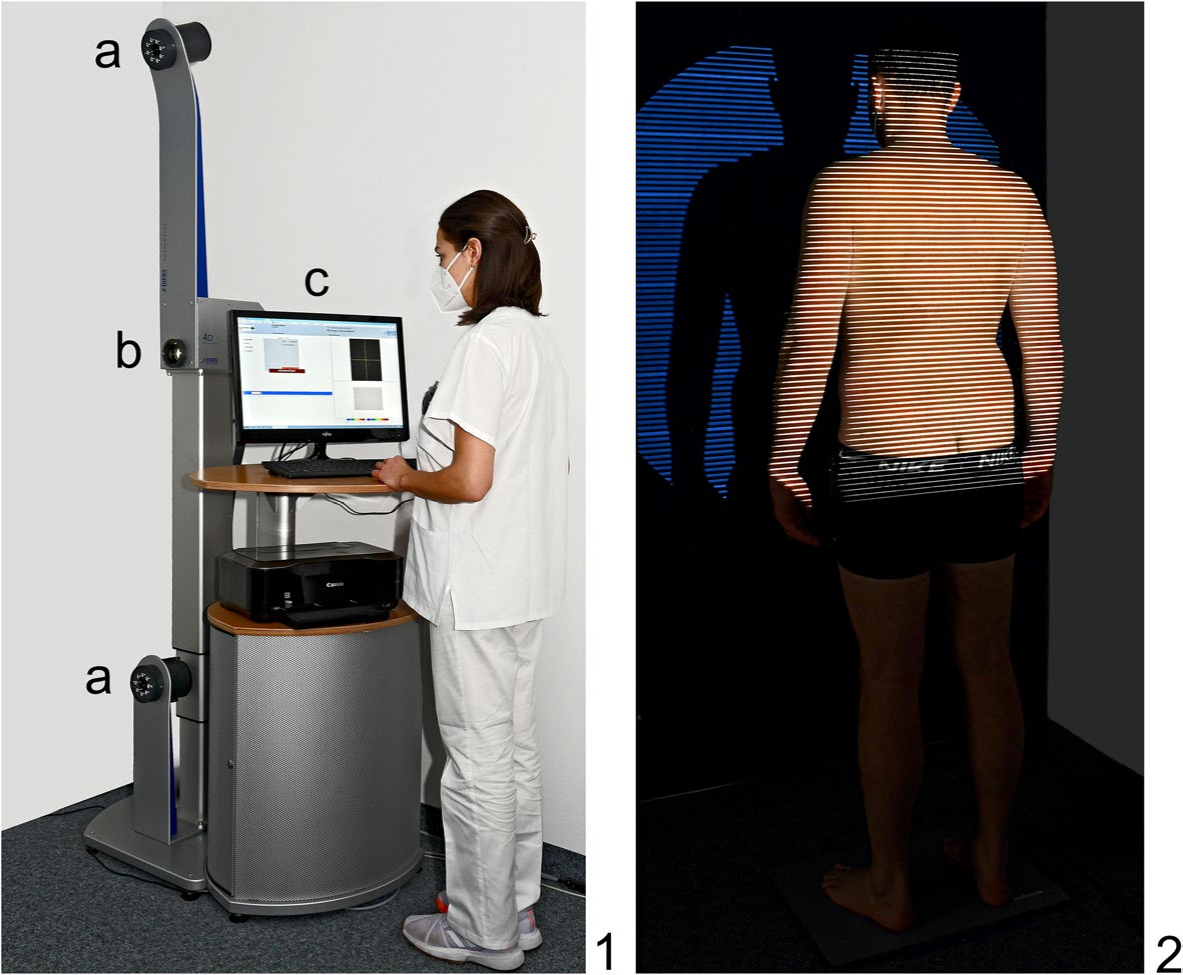
Diers Formetric 4D equipment: (**a**) Camera units for image capture. **b** Projector which generates the line grid projection. **c** Workstation incorporating the software DiCam v2.5.15 (DIERS International GmbH; Schlangenbad, Germany). The image-captured line grid

**Fig. 2:**
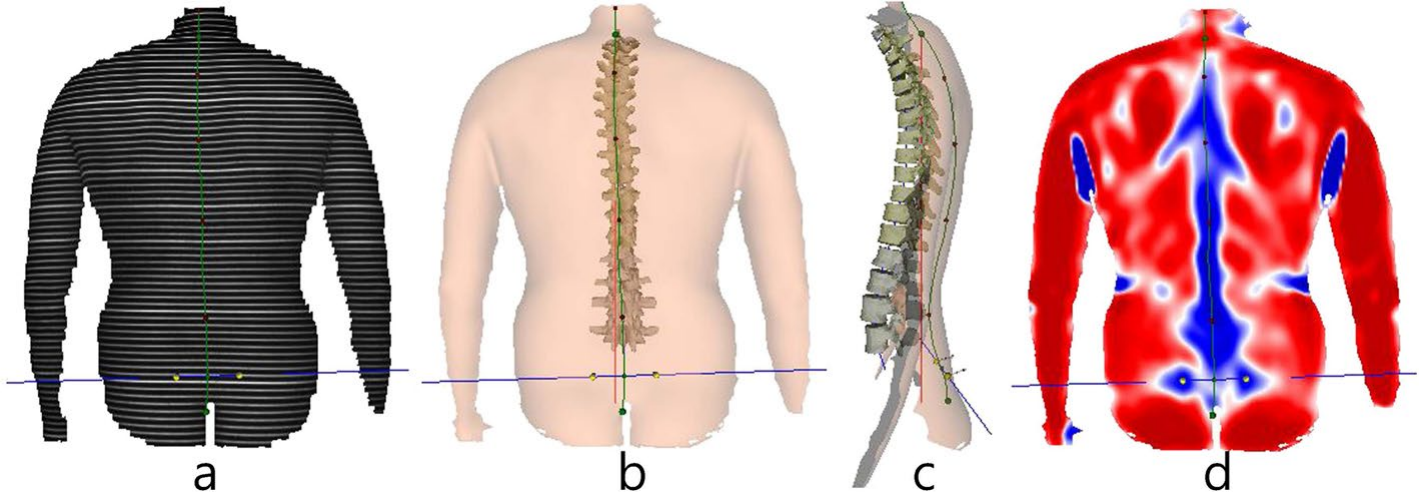
Image examples generated by DiCam v2.5.15: (**a**)The image-captured line grid. **b** Reconstructed three-dimensional model of the dorsal view of the spine. **c** Reconstructed three-dimensional model of the lateral view of the spine. **d** Convex (red) and concave (blue) curvatures

Patients were measured using the Diers Formetric 4D system with DiCam v2.5.15 software (DIERS International GmbH; Schlangenbad, Germany) (Fig. 1) in the 4D average modality at each of three different time points (at baseline, after the first therapy unit, and after completion of therapy). A 3D analysis of the spine and pelvis was performed using the projector. The measurement technique was based on the principle of triangulation and used a grid projected by the DIERS Formetric 4D projector onto the dorsal surface of the patient (Fig. 1). Using two cameras (Fig. 1), the curvature of individual lines was captured and a 3D image of the patient's surface was calculated by the computer software by triangulation. Triangulation is a measurement technique that performs optical distance measurements by measuring angles within a triangle with a known base length. Anatomical fixed points (Table 2), the spinal midline, and spinal rotation are automatically recognized by the software, and a correlation model of the body statics is calculated. Based on the detected reference points (Table 3), the mean surface rotation is determined and displayed in colour (Fig. 2). Blue areas correspond to concave structures, and red to convex curvatures. The system then creates a 3D reconstruction of the spine and pelvis based on its calculations (Fig. 2).

**Table 2:** Anatomic landmarks automatically detected by DiCam v2.5.15

Abbreviation	Anatomic Landmarks
VP	Vertebra prominens
SP	Sacrum Point
DL	Left lumbar dimple
DR	Right lumbar dimple

**Table 3:** Additional relevant anatomic points and lines detected by DiCam v2.5.15

Abbreviation	Anatomic landmarks and lines
DM	Midpoint of lumbar dimples
CA	Cervical apex
KA	Kyphotic apex
LA	Lordotic apex
ICT	Cervicothoracic junction
ITL	Thoracolumbar junction
ILS	Junction between lumbar lordosis & sacrum
Perpendicular line	Perpendicular line through the kyphotic apex

To obtain the fourth dimension, a time component is also recorded. For this purpose, a series of twelve images is recorded and evaluated within 6 s; the images are then averaged by the software. The individual variables are then calculated on the basis of these results. The DIERS Formetric 4D back scanner records 84 variables, 10 of which were used for data evaluation in this study. The patient scans were performed by the study supervisors under standardized conditions.

### Measuring procedure

All patients were initially measured before starting their first physiotherapeutic treatment to analyse their individual baseline condition (T1 = 1st baseline MT). To ensure the standardization and reproducibility of the measurements, the same procedure was consistently followed. The measurement was performed with the subject in a standing position in a darkened room without disturbing light sources. The test persons positioned themselves hip-width apart on the DIERS pedo-scan plate with their upper body uncovered. The subject faced away from the projector so that the DIERS Formetric 4D could visually scan the subject's back. Prior to analysis, were prepped to expose the hairline, and jewellery was removed to avoid potential reflections. In addition, the subjects were asked to adopt a relaxed habitual posture, to let their shoulders droop and, if possible, not to move and to continue breathing calmly during the recording time of 6 s. The same methodology was used for all other back scans taken during the course of the study.

The second back scan (T2 = 2nd MT scan) was taken right after the first therapy session according to the standardized procedure. It was important that the patient did not bite during the time between getting up from the therapy bench and the completion of the scan. This procedure was chosen in order to obtain the most unaltered values of the variables possible in the second scan. The third and final scan (T3 = 3rd MT scan) was performed after completion of therapy, using the same standardized procedure as for the previous scans.

Parallel to the participating TMD patients, control data were collected from subjects without a TMD diagnosis. The control subjects were scanned twice using the DIERS Formetric 4D. The first scan (T1 = 1st control baseline) took place to determine the baseline condition. The second scan (T2 = 2nd control scan) was performed after an average of 1 year and compared with the baseline scan. The projection was performed as previously described for the study patients. The control data collection was necessary to see if significant differences in the acquired variables between two scans could be detected in the treated TMD patients as well as in the control patients.

### Variables

For 3D monitoring of both our patients and control subjects over time, the following 10 variables were recorded and analysed at each scan appointment: in the sagittal plane, the kyphotic angle, lordotic angle, flèche cervicale, flèche lombaire, kyphotic apex, trunk tilt, and pelvic tilt; in the frontal plane, the perpendicular deviation; and in the transverse plane, the pelvic torsion and surface rotation (Table 4). Of these, the kyphotic angle, flèche cervical and kyphotic apex relate to the upper spine; the lordotic angle, flèche lombaire, pelvic torsion, and pelvic tilt to the lower spine; and the perpendicular deviation, trunk inclination, and surface rotation to the torso. These variables were selected to measure posture in three planes and posture from head to pelvis.

**Table 4:** The 10 investigated spine and posture variables from the Formetric 4D examination

Variable	Definition
Perpendicular deviation (mm)	Angle between the connecting line VP-DM and a centroidal axis through VP
Kyphotic angle (°)	Angle measured between the tangents at VP and at ITL
Lordotic angle (°)	Angle measured between the tangents at ITL and at DM
Flèche cervicale (mm)	Distance from the cervical apex to the perpendicular line
Flèche lombaire (mm)	Distance from the lordotic apex to the perpendicular line
Kyphotic apex (mm)	Distance between kyphotic apex and VP-DM
Trunk inclination (mm)	Angle between the connecting line of VP-DM and a centroidal axis
Pelvic tilt (°)	Height difference between DL and DR in the frontal plane
Pelvic torsion DL-DR (°)	The torsion of the surface normal plane touching both lumbar dimples
Surface rotation (°)	Maximal surface rotation to the right (+ max)/and left (− max), which corresponds to the spinous process line in the transversal plane

### Statistical analysis

To analyse how the variables changed between the measurement times, the absolute values of the differences between two scans were calculated. For example, control group│T2 − T1│ compares the second scan of the control group with the first scan of the control group. The statistical significance of the differences between the control and MT groups was determined using the Mann–Whitney U test. A significance level of 0.05 was set.

## Results

A total of 30 subjects were included in the study: 15 TMD patients (7 women, 8 men) and 15 control subjects (8 women, 7 men). There was a slight difference between the groups in terms of mean age (TMD patients: 37.8 years, age range: 24–79 years; control group: 26.5 years, age range: 23–35 years) and no significant difference between the two groups in terms of male to female ratio (8:7 and 7:8, respectively). Therefore, the groups had similar characteristics in terms of sex and slightly different characteristics in terms of age. All treated patients underwent the first and second measurements, participated in all three treatments, and also underwent the third measurement. Similarly, all control subjects were also able to undergo the first and second measurements.

The mean values and standard deviations of the absolute values of the differences of two scans for the analysed posture parameters in the course of manual therapy for the temporal comparisons of the baseline first scan T1 (before the start of therapy), the second scan T2 (after the first therapy session or after 6 months for the control subjects), and third scan T3 (after the last therapy session), averaged over the 15 subjects/patients are shown in Table 5.

**Table 5:** Mean values (with standard deviations) averaged for the 15 MT subjects for the 10 investigated variables compared with those of the control group

Comparison of measurement times	Mean (SD)			
	MT│T2–T1│	MT │T3–T1│	MT │T3–T2│	Control│T2–T1│
Perpendicular deviation (mm)	8.432 (8.946)	7.974 (7.708)	9.055 (11.152)	6.127 (4.368)
Kyphotic angle (°)	5.361 (5.398)	5.643 (5.335)	3.985 (2.499)	3.547 (2.927)
Lordotic angle (°)	4.871 (4.134)	3.021 (3.111)	6.163 (6.359)	4.565 (5.147)
Flèche cervicale (mm)	17.041 (24.439)	17.075 (28.524)	6.397 (5.803)	9.288 (6.965)
Flèche lombaire (mm)	4.202 (2.153)	5.804 (5.130)	6.071 (6.353)	5.557 (4.569)
Kyphotic apex (mm)	11.946 (13.682)	11.538 (16.790)	11.538 (16.790)	15.629 (14.084)
Trunk inclination (mm)	10.517 (8.791)	12.007 (9.163)	15.081 (14.437)	12.556 (10.025)
Pelvic tilt (°)	5.388 (6.679)	6.839 (6.506)	7.581 (9.163)	3.009 (3.301)
Pelvic torsion (°)	1.979 (2.093)	2.169 (1.917)	1.876 (1.734)	2.844 (1.976)
Surface rotation (°)	2.821 (3.423)	1.881 (2.493)	3.098 (3.778)	1.041 (0.964)

The results for the statistical analysis presented in Table 6 were adjusted according to the Mann–Whitney U test.

**Table 6:** p-values (p-values ≤0.05 in bold) of the Mann-Whitney U-test for the 10 investigated parameters as difference of the measurement times comparing control group & physiotherapy group

Measurement times in comparison between control and physio group	***p*-value**		
	Control│T2–T1│to MT│T2–T1│	Control│T2–T1│to MT│T3–T1│	Control│T2–T1│to MT│T3–T2│
Perpendicular deviation	0.983	0.787	0.835
Kyphotic angle	0.683	0.202	0.389
Lordotic angle	0.683	0.539	0.412
Flèche cervicale	0.902	0.653	0.161
Flèche lombaire	0.52	0.838	0.967
Kyphotic apex	0.217	**0.029**	**0.037**
Trunk inclination	0.624	0.838	0.838
Pelvic tilt	0.461	0.067	**0.029**
Pelvic torsion	0.067	0.148	0.135
Surface rotation	0.389	0.372	**0.049**

There were no significant differences in trunk inclination, pelvic torsion, flèche cervicale, flèche lombaire, kyphotic angle, lordotic angle, and perpendicular deviation between the control subjects and the study participants with TMD.

**Fig. 3:**
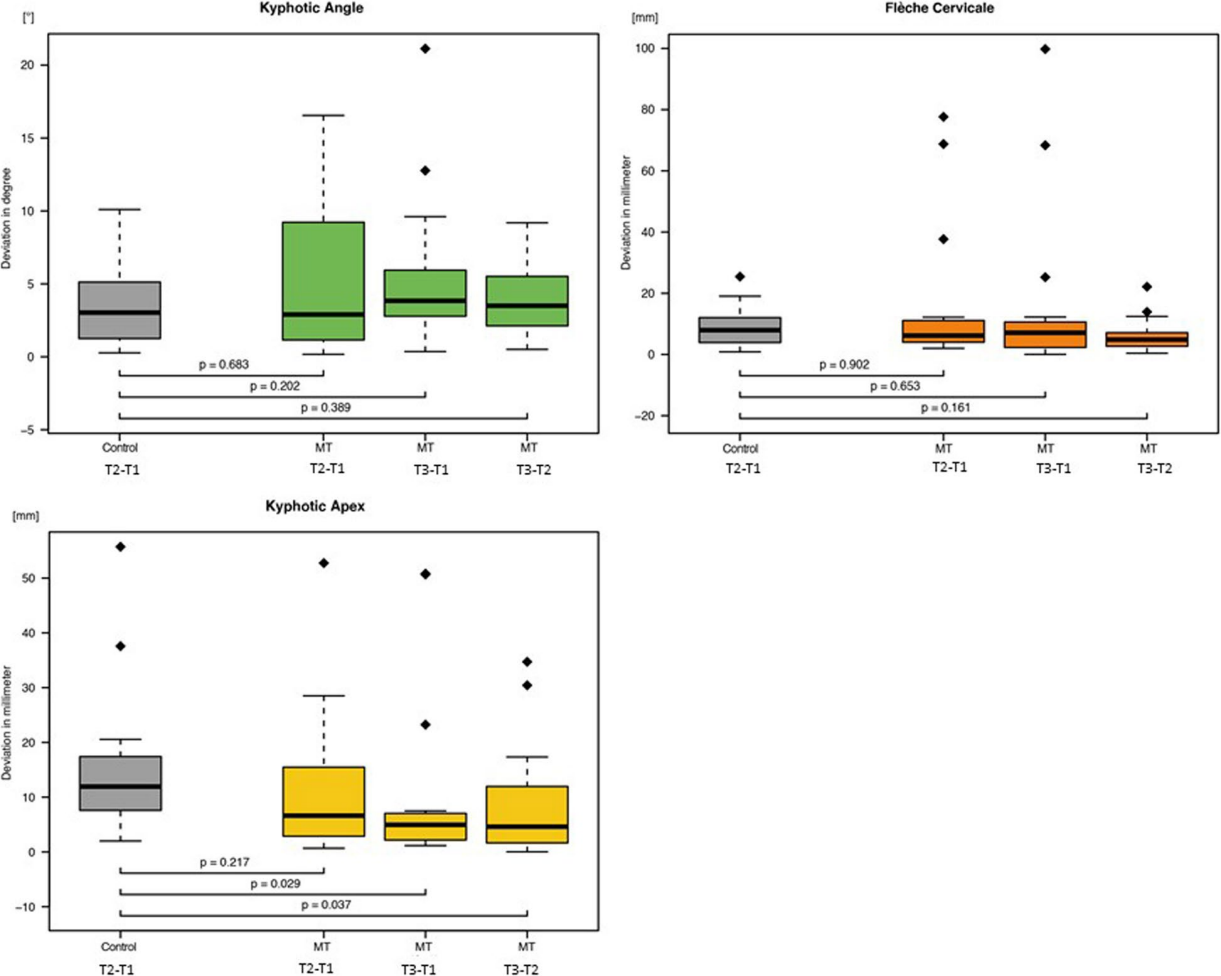
Boxplots of the three investigated upper spine parameters (See Tab. 2 for abbreviations)

**Fig. 4:**
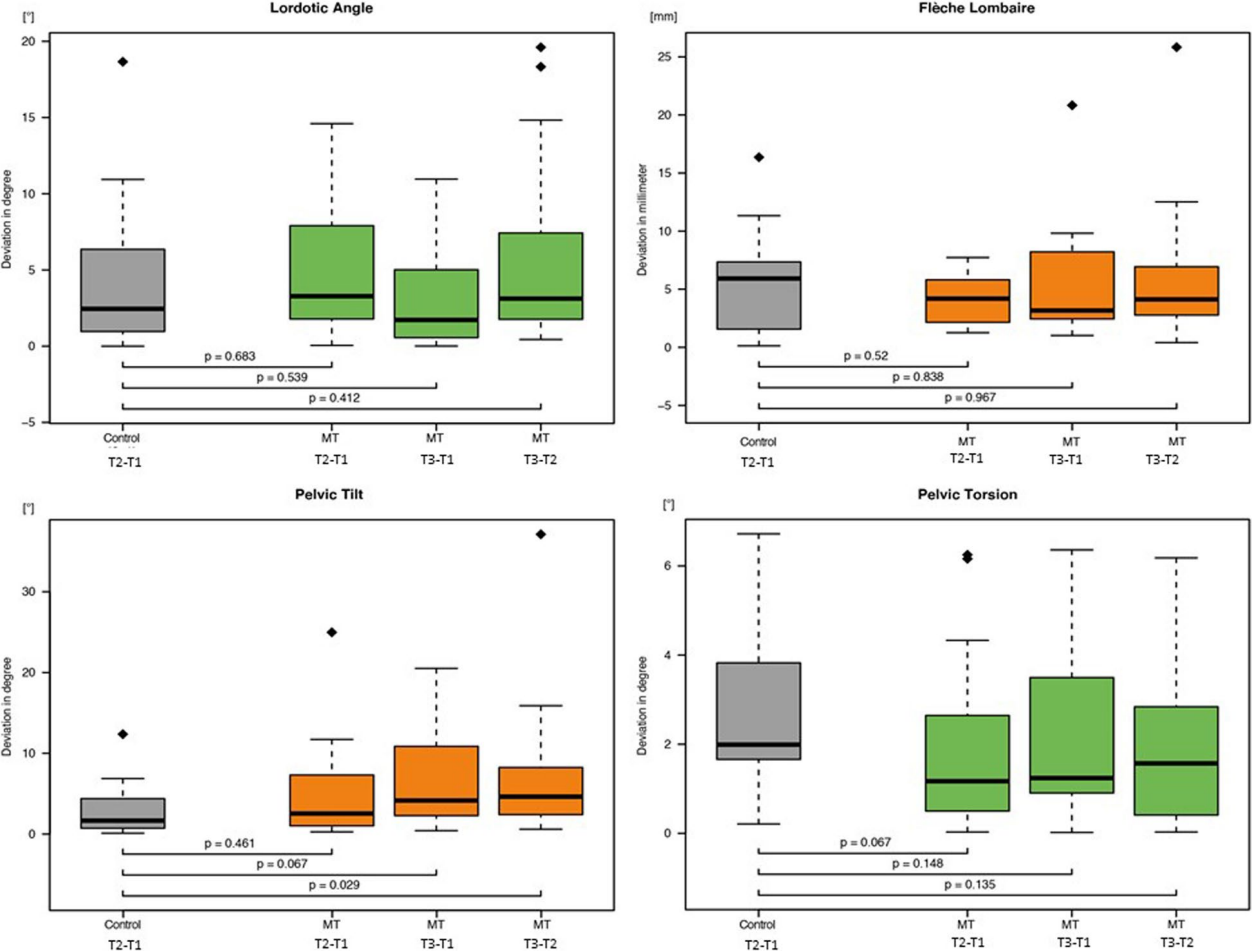
Boxplots of the four investigated lower spine parameters (See Tab. 2 for abbreviations)

**Fig. 5:**
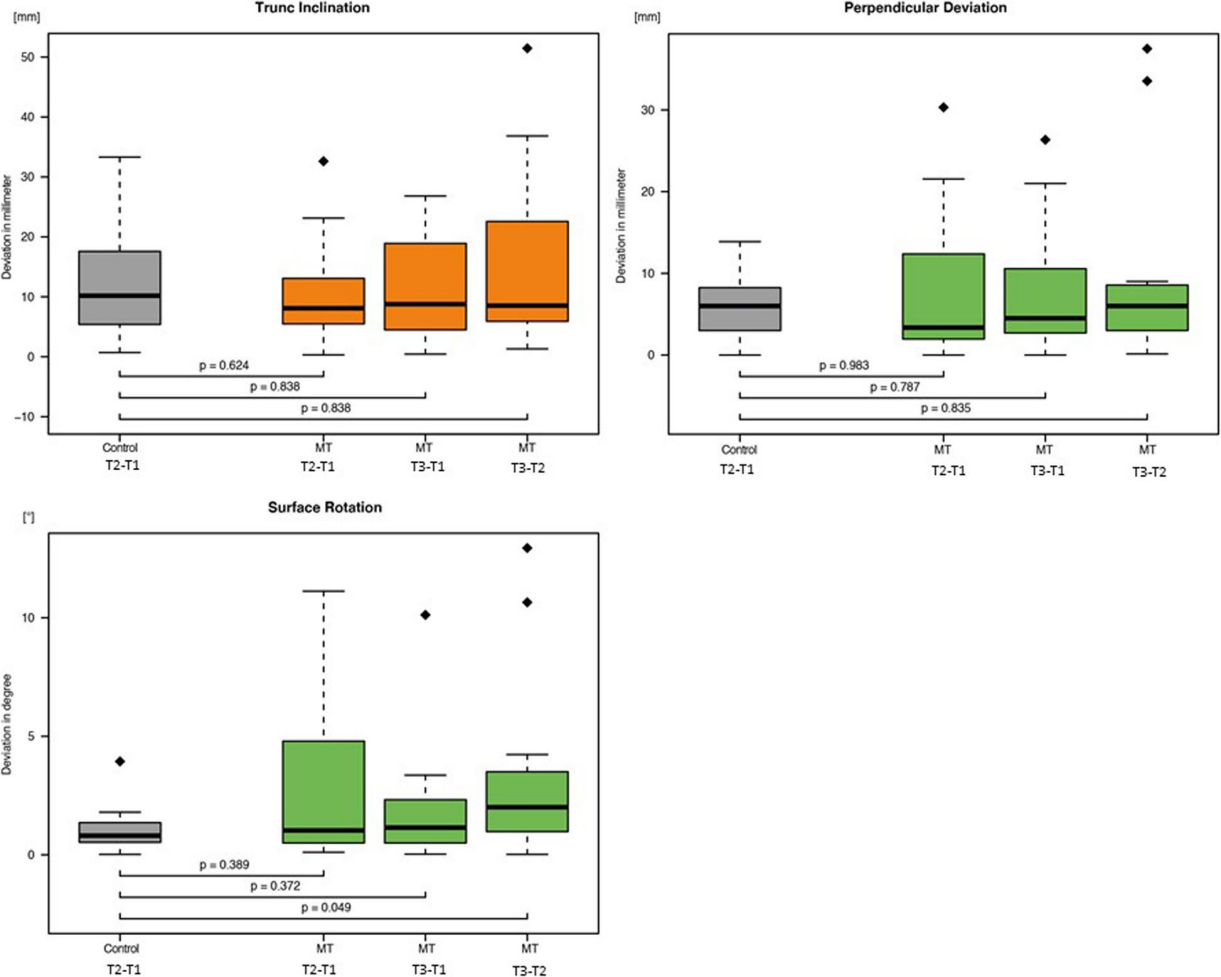
Boxplots of the three parameter of the torso (See Tab. 2 for abbreviations)

Statistically significant differences were found in the upper spine (Fig. 3), lower spine (Fig. 4), and torso variables (Fig. 5). These differences were in the pelvic tilt, surface rotation, and kyphotic apex.

When the control│T2 − T1│was compared with MT│T3 − T2│, the surface rotation, pelvic tilt, and kyphotic apex measurements showed significant changes (*p* = 0.049, *p* = 0.029, and *p* = 0.037, respectively). The kyphotic apex differed significantly between control│T2 − T1│and MT│T3 − T1│ (*p* = 0.029).

## Discussion

The aim of the present study was to use video rasterstereography to analyse the effect of MT on posture in TMD patients in comparison with control subjects. For this purpose, ten different postural parameters in 15 TMD patients and 15 control subjects were recorded by means of rasterstereography at different time points and compared with each other. Comparing the physiotherapy-treated patients with untreated control subjects, significant postural differences were found when comparing the first with the second treatment session.

In the current literature, there is a lack of studies that clearly demonstrate a correlation between TMD and posture [4, 59, 76], although there is a widely recognized relationship between TMD and other craniocervical complaints, such as headache or neck pain. This can be explained by the anatomical, functional, and neurophysiological relationship between the TMJ and the upper cervical spine [77, 78]. Establishing a causal relationship between posture, TMJ disorders, and the influence of manual therapy would improve our understanding and treatment of temporomandibular disorders [61, 79].

In this study, we analysed 3D posture and spine conformation by using rasterstereography, which has been established as a reliable and user-friendly method for the assessment of whole-body posture [80–82]. Betsch et al. and Degenhardt et al. have shown that measurements acquired through rasterstereographic imaging with the DIERS Formetric 4D system are reliably validated and reproducible, and that dynamic spinal posture is mapped with sufficient accuracy [56, 83]. The applied 4D methodology has been shown to provide reliable results in regard to both intra-day and inter-day measurements [57]. In examinations of scoliosis patients, changes in standard radiographs correlated with those obtained from rasterstereography [84]**.**

Conservative treatment methods such as MT are part of the primary treatment level of non-invasive TMD therapy. MT is a specialization within physiotherapy and has been successfully used as a treatment method for spinal disorders, among others [85, 86]. MT is used to reduce pain, relax muscles, improve ROM, release adhesions, and reduce local ischemia [87]. Several systematic reviews have demonstrated a positive effect of MT as a sole therapeutic modality or in combination with other therapeutic techniques for TMD treatment [33, 34, 88]. In their studies, Ferreira et al. and Grondin et al. showed that patients with TMD have limited mobility of the cervical spine [89, 90]. Targeted to the cervical spine and TMJ, this form of therapy for TMD was able to produce positive effects in patients' pain perception and TMJ ROM in Tuncer et al. [52]. The review by La Touche et al. demonstrated better efficacy of cervical MT for short-term pain relief and functional improvement when applied to the cervical spine and craniomandibular system rather than only to the cervical spine. This is justified by the neurophysiological and anatomical connections between these two areas [50].

Some studies have shown that there is a relationship between posture and TMD and that MT can influence this constellation [3, 62, 73, 91, 92], whereas other studies could not confirm this relationship [2, 93, 94]. The results of the present study show a significant influence of MT on posture in TMD patients during MT therapy as compared with a control group.

Lippold et al. were able to demonstrate a correlation between craniofacial morphology and posture using rasterstereography. Not only were changes in the cervical spine found, but also adjustments down to the lower spinal segments [95]. A similar effect was found in the current study due to the significant changes in pelvic torsion. These results contradict the findings of Visscher et al., who refuted a correlation between TMD and altered head posture [5]. Data from other studies were able to confirm that poor posture affects the musculature and, due to the functional unity of the stomatognathic system, the mandibular position is likewise modified, which can result in TMJ dysfunction and thus TMD [61, 62, 92]. In their study, Marz et al. demonstrated spinal changes in flèche cervicale, flèche lombaire, and the kyphotic angle at different occlusal positions using rasterstereography. These changes correspond to postural adjustments up to the thoracic spine [96]. Therefore, there is growing interest in the relationship of TMD to posture.

In this study, changes in postural and spinal variables in TMD patients were induced by the application of MT to craniocervical structures over the prescribed period of MT and recorded using rasterstereography. After comparing 10 spinal and postural parameters (Table 4) at three and two different time points, respectively (Table 5), between a control group and a physiotherapy group, changes in three variables were found to be statistically significant (Table 6). Thus, postural profile changes in TMD patients in the course of MT treatment could be detected and should not be underestimated. The null hypothesis was rejected.

The mean deviations for the surface rotation parameter at MT│T2 − T1│ and MT │T3 − T1│ were 2.821 and 1.881, respectively. However, the interpretation of this variable is affected by the high standard deviation. The pelvic tilt and kyphotic apex parameters also have similarly high deviations, making interpretation of those results difficult as well. No significance could be established after termination of the physiotherapeutic treatment. None of the other evaluated variables showed statistically significant differences. Possible explanations for the time-limited changes that were identified include patients regressing to their long-established postural patterns as well as neuromuscular compensation to maintain body balance, which may have masked possible changes after therapy ended.

While interpreting the results, the following limitations were identified that should be considered. The lack of significant differences in some variables may be attributed to several factors, as appropriate. First, the patients were a heterogeneous sex-nonspecific group with a wide age range; thus, age- and sex-specific habitual postural differences cannot be excluded. Second, only a small group of patients was included in our pilot study. We noted changes in some parameters—for example, kyphosis and lordosis in both directions, flexion, and extension—without establishing a preference for one direction. This suggests that a change in one direction cannot be predicted or that the sample in the present study was too small to detect significant postural differences in this spinal segment. The short screening test by Jakstat and Ahlers that we use to identify potential TMJ disorders do not differentiate between occlusopathies, arthopathies, and myopathies. Patients whose TMD is not caused by muscular imbalances or ongoing myofacial chains may benefit less from the manual therapy method used. This may also be the cause of less measurable changes in posture Further studies with a larger sample could possibly elicit a probability of spine tilt in a particular direction and should differentiate the various subgroups of TMD even more precisely in order to avoid this limitation. Further studies with a larger sample could possibly elicit a probability of spine tilt in a particular direction. Third, the unambiguous reproducibility of the recording position was achieved by standardized positioning of the subjects. This is absolutely necessary for the collection of the variables perpendicular deviation and trunk inclination. However, due to the standardization process, the subjects could have switched from their natural habitual posture to a corrected one, which was then also measured during the recording instead of the habitual spinal posture. Fourth, the fixed points (Table 2) vertebra prominens (VP) and dimple left/right (DL/ DR), which are relevant to the variable determination according to the manufacturer's specifications, are usually correctly determined by the DIERS Formetric. An incorrect assignment of these points, however, can make a fixed-point correction by the exposure supervisor necessary. Since the reliability and accuracy of the parameter calculation strongly depends on the fixed-point determination, individual manual corrections of the fixed points may lead to deviations in their calculation. In addition, the spine and posture parameters were determined using rasterstereography based on anatomical landmarks; direct measurement or analysis of radiographs could lead to different results. Fifth, the upper part of the cervical spine in particular, which was expected to show the greatest alterations, was less precisely imaged with the DIERS Formetric than was the rest of the spine, complicating postural analysis in this region. Sixth, it is important to consider that the study period and the amount of MT prescribed may have been too short to demonstrate significant changes in posture. Additional studies could investigate whether a longer treatment period would alter the results. Seventh, the duration of TMD complaints and the associated pain intensity could also play a role. Due to the large age range present, there may be large differences between patients, especially in TMD duration.

## Conclusion

Postural change mostly occurred after the first therapy session, while at the end of therapy most patients compensated back to their initial posture. The postural changes could be observed not only in areas close to the TMJ, but were continuous to the pelvis. Based on the present results and with the limitations mentioned, we would consider MT as a supportive therapy for TMD patients.

## Data Availability

The datasets generated and/or analysed during the current study are not publicly available due as only a declaration of consent from the subjects for the statistical analysis and publication of the present work is available, but no generalized declaration of consent for the patient-related raw data. However, these are available on reasonable request from the corresponding author**.**

## Data availability

No datasets were generated or analysed during the current study.
